# Supplementary material for: The basic helix-loop-helix transcription factor SHARP1 is an oncogenic driver in MLL-AF6 acute myelogenous leukemia
Source: Nat Commun. 2018 Apr 24;9:1622. doi: 10.1038/s41467-018-03854-0 (PMC5915391; doi:10.1038/s41467-018-03854-0)
Supplement: Supplementary file 1 — Supplementary information [file 41467_2018_3854_MOESM1_ESM.pdf]

The basic helix-loop-helix transcription factor SHARP1 is an oncogenic driver in MLL-AF6 Acute Myelogenous Leukemia

Numata *et al.*

Supplementary figures 1 – 8

Supplementary tables 1 – 5

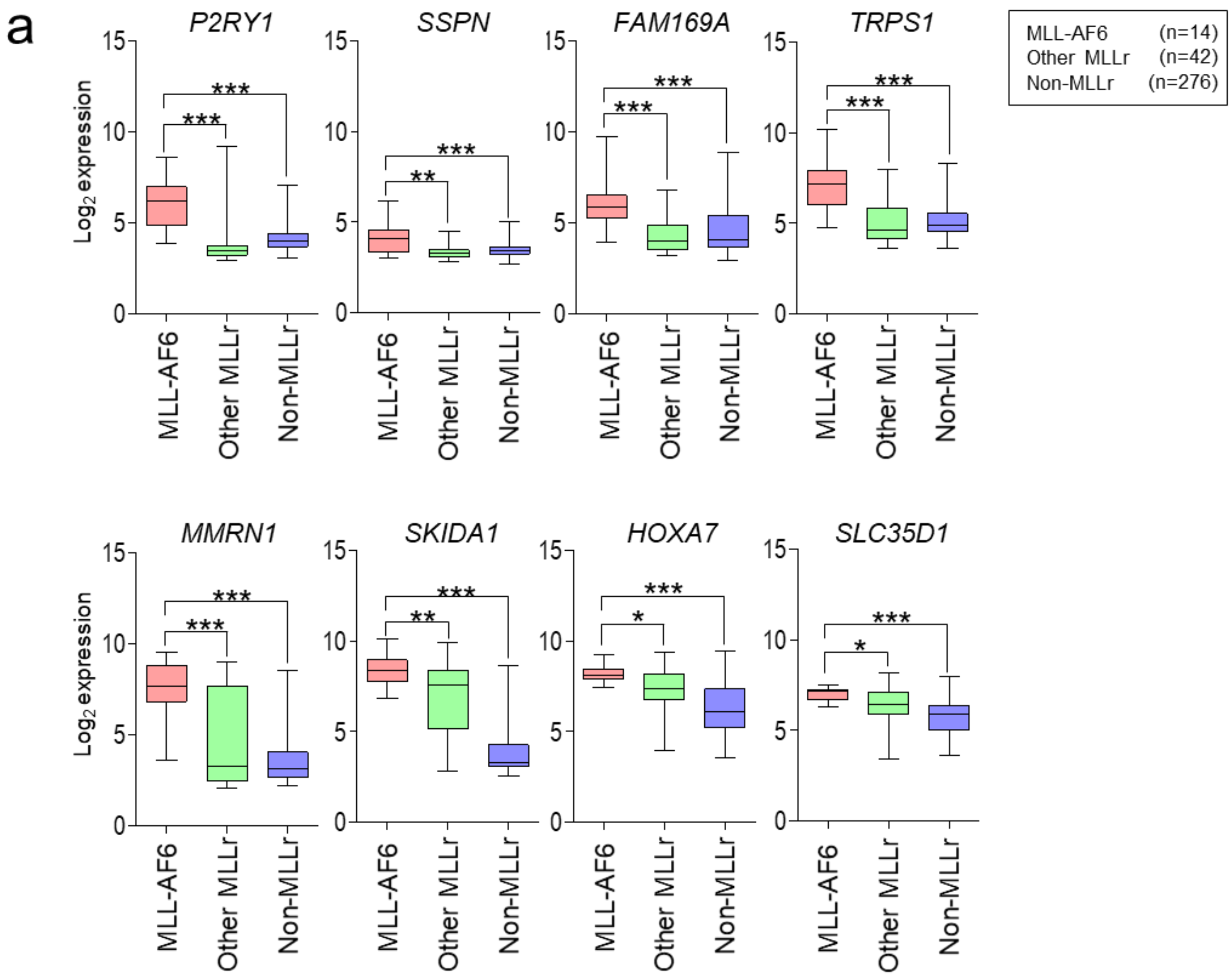
a
P2RY1
SSPN
FAM169A
TRPS1
MMRN1
SKIDA1
HOXA7
SLC35D1
Log2 expression
MLL-AF6
Other MLLr
Non-MLLr
MLL-AF6 (n=14)
Other MLLr (n=42)
Non-MLLr (n=276)

Numata et al., Supplementary Figure 1

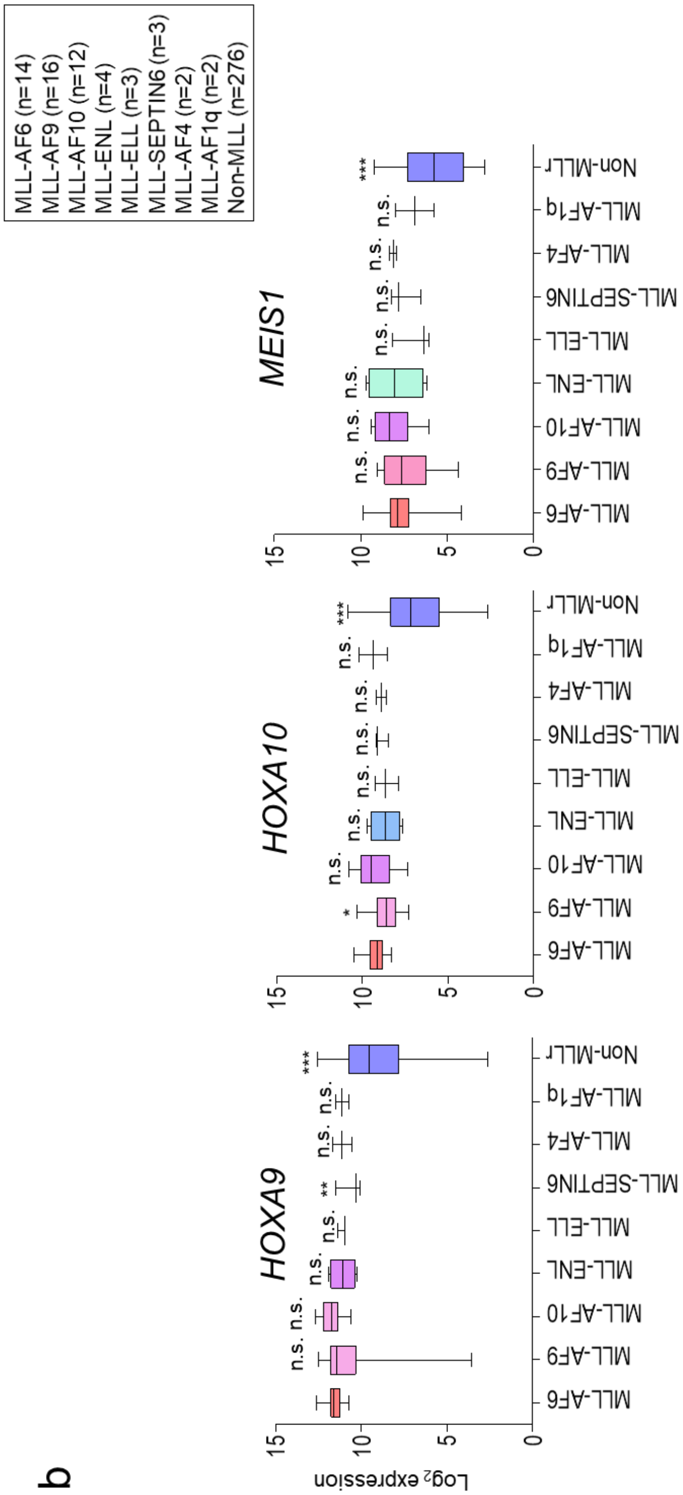

Supplementary Figure 1. Related to Figure 1.

(a) Box plots showing expression of MLL-AF6 specific target genes in AML patients. (b) Box plots showing expression of MLL-AF6 specific targets (SHARP1, P2RY1, and TRPS1) and the canonical target genes of MLL-FPs (HOXA9, HOXA10, and MEIS1) in AML patients according to the subtypes. Gene expression data of patients were obtained from GSE19577, GSE14468 and GSE61804. All box plots extend from the 25th to 75th percentiles and the whisker extends from the minimum level to the maximum. Median value is plotted in the box. *P* values are calculated based on the comparison between each subtype and MLL-AF6. $*p < 0.05$, $** p < 0.01$, $***p < 0.001$

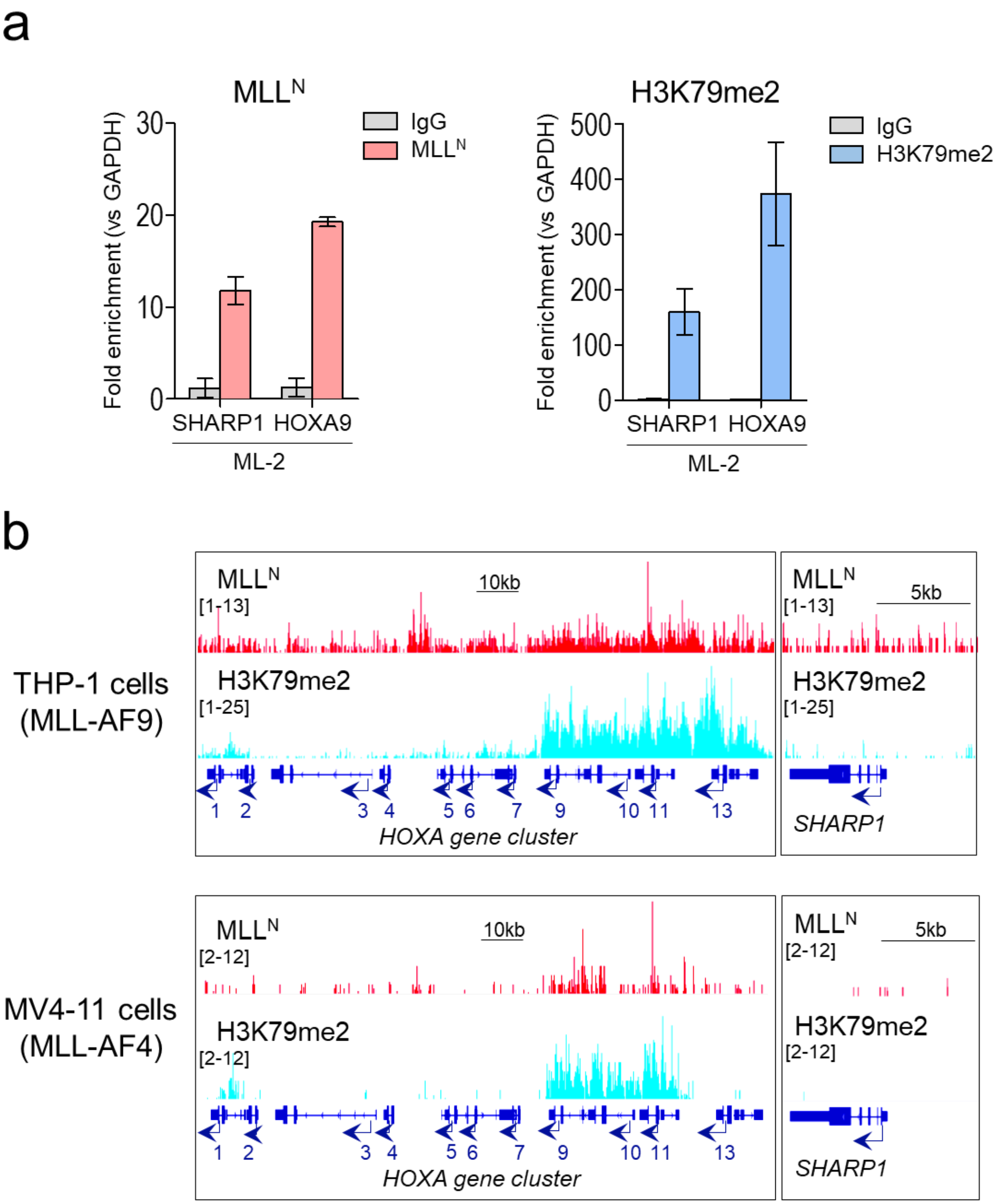

Supplementary Figure 2, Related to Figure 2

(a) MLL^N and H3K79me2 ChIP-qPCR in the *SHARP1* and *HOXA9* promoters, normalized to GAPDH promoter in the ML-2 cells (MLL-AF6). Data are from three independent experiments and presented as mean ± s.e.m. (b) MLL^N ChIP-seq profiles of THP-1 (MLL-AF9) (top panel) and MV4-11 cells (MLL-AF4) (bottom panel) at the loci of the *HOXA* gene cluster and *SHARP1* gene. ChIP-seq data were obtained from GSE79899.

a

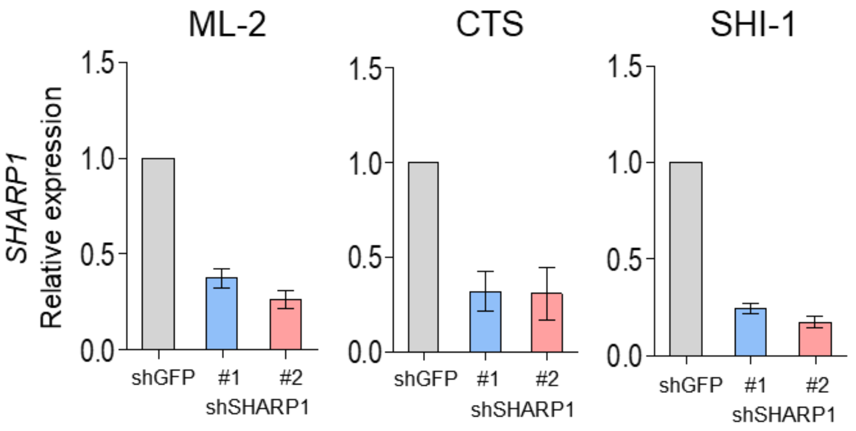
ML-2
CTS
SHI-1
SHARP1
Relative expression
1.5
1.0
0.5
0.0
shGFP
#1
#2
shSHARP1

b

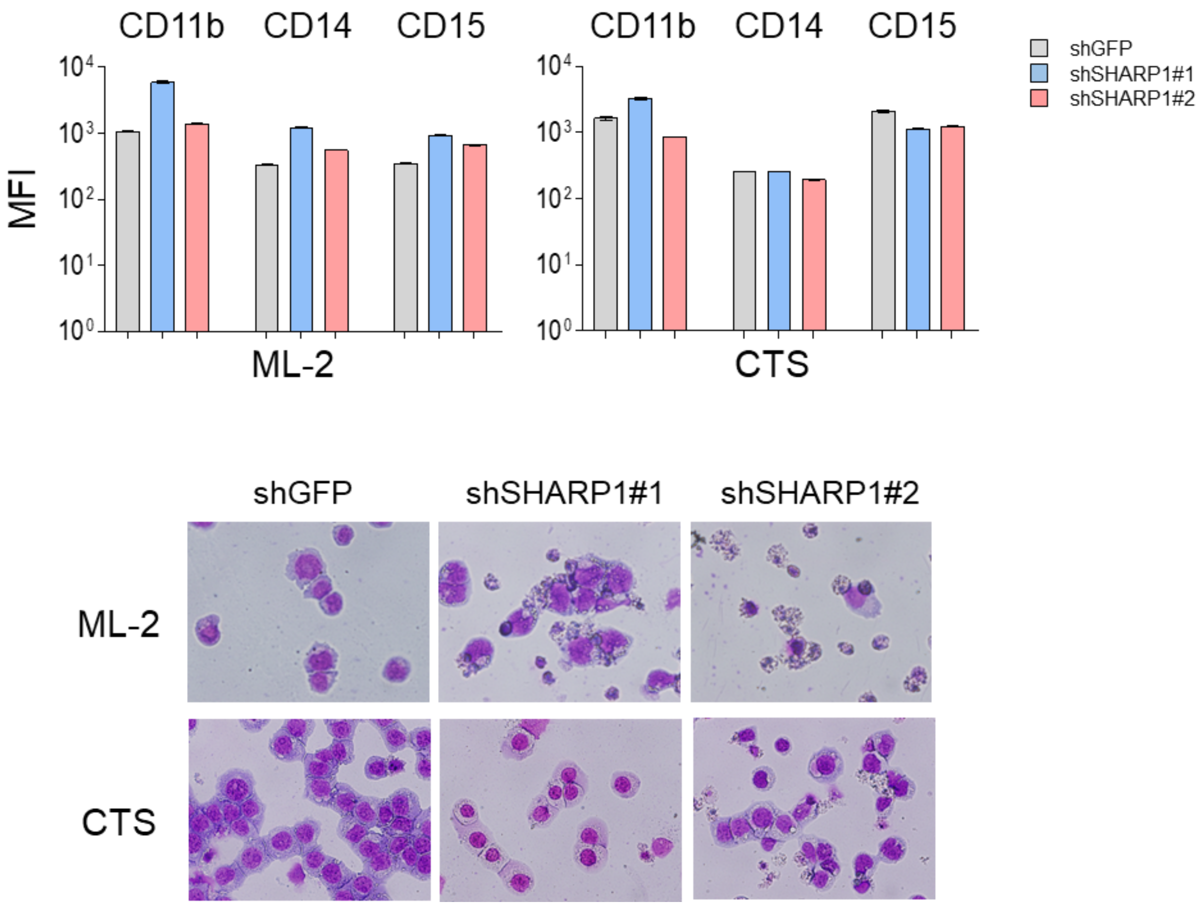
CD11b
CD14
CD15
MFI
10^4
10^3
10^2
10^1
10^0
ML-2
CTS
shGFP
shSHARP1#1
shSHARP1#2

c

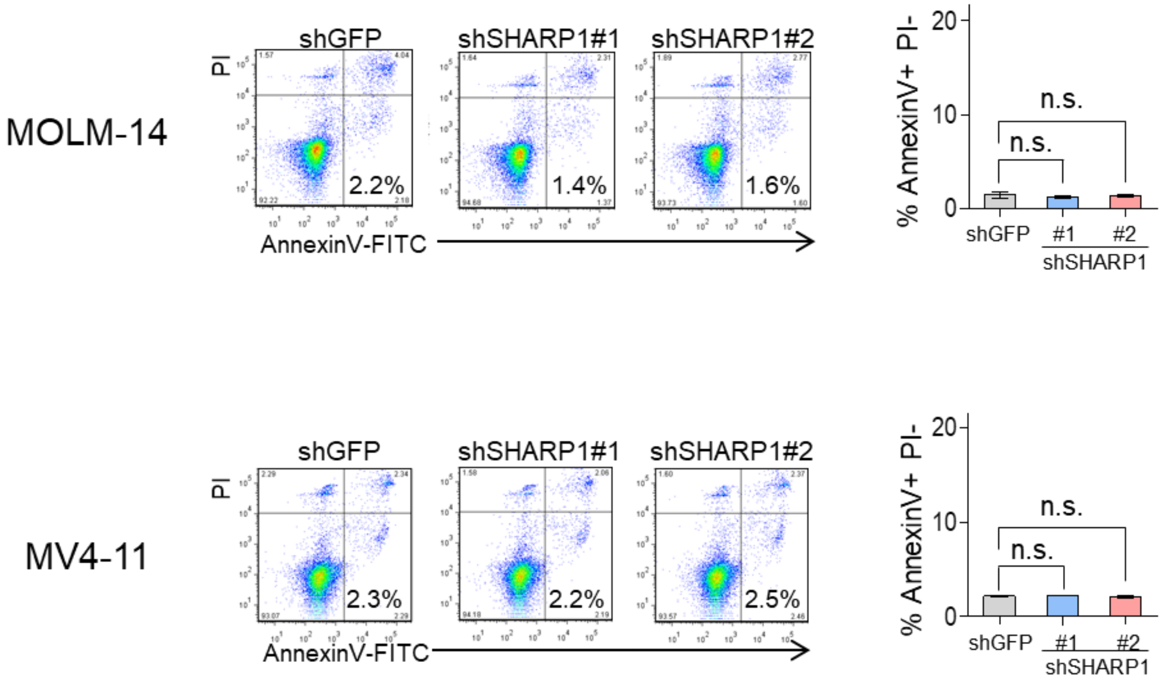
MOLM-14
MV4-11
shGFP
shSHARP1#1
shSHARP1#2
PI
AnnexinV-FITC
2.2%
1.4%
1.6%
2.3%
2.2%
2.5%
% AnnexinV+ PI-
n.s.
shGFP
#1
#2
shSHARP1

d

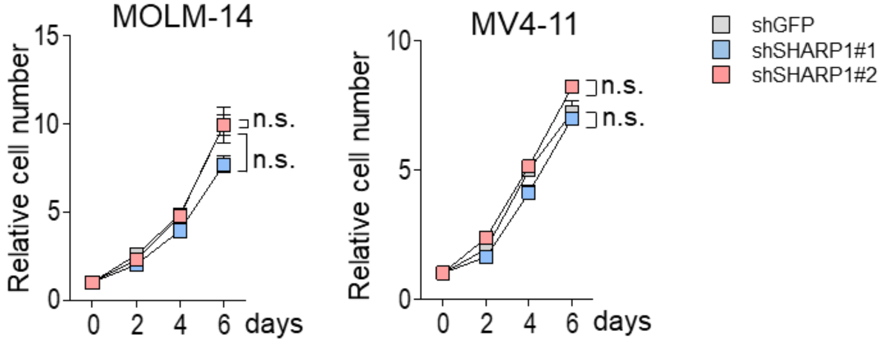
MOLM-14
MV4-11
Relative cell number
days
n.s.
shGFP
shSHARP1#1
shSHARP1#2

e

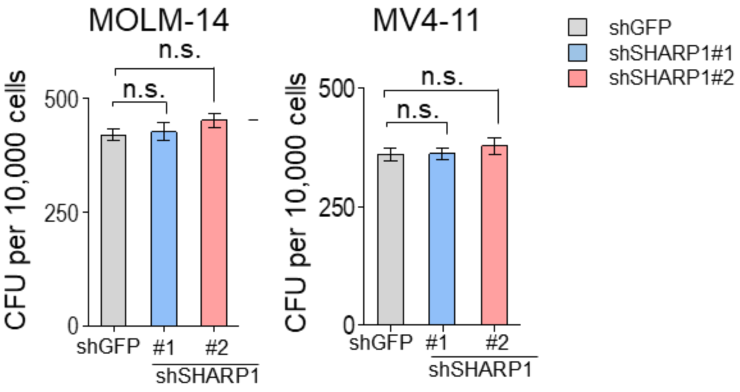
MOLM-14
MV4-11
CFU per 10,000 cells
n.s.
shGFP
#1
#2
shSHARP1
shGFP
shSHARP1#1
shSHARP1#2

Supplementary Figure 3. Related to Figure 3

(a) qPCR for SHARP1 mRNA in ML-2, CTS and SHI-1 cells transduced with the indicated shRNAs. Expression is the value relative to shGFP. (b) Expression levels of mature granulocytic and monocytic markers, CD11b, CD14 and CD15, measured by FACS in ML-2 and CTS cells transduced with the indicated shRNAs (top panel). Giemsa staining of ML-2 and CTS cells transduced with the indicated shRNAs (bottom panel). (c) Representative AnnexinV and PI FACS plot and percentage of AnnexinV+ and PI- cell of MOLM-14 cells (top panel) and MV4-11 cells (bottom panel) transduced with the indicated shRNAs. (d) Cell count of MOLM-14 cells (left panel) and MV4-11 cells (right panel) transduced with the indicated shRNAs in culture. The value is determined as fold increase in cell number relative to the number of cells initially plated. (e) Colony-forming units (CFU) per 10,000 cells of MOLM-14 cells (left panel) and MV4-11 cells (right panel) transduced with the indicated shRNAs, with the number of colonies observed 7 days after the plating. The graphs are representative examples of three independent experiments and presented as mean ± s.e.m.

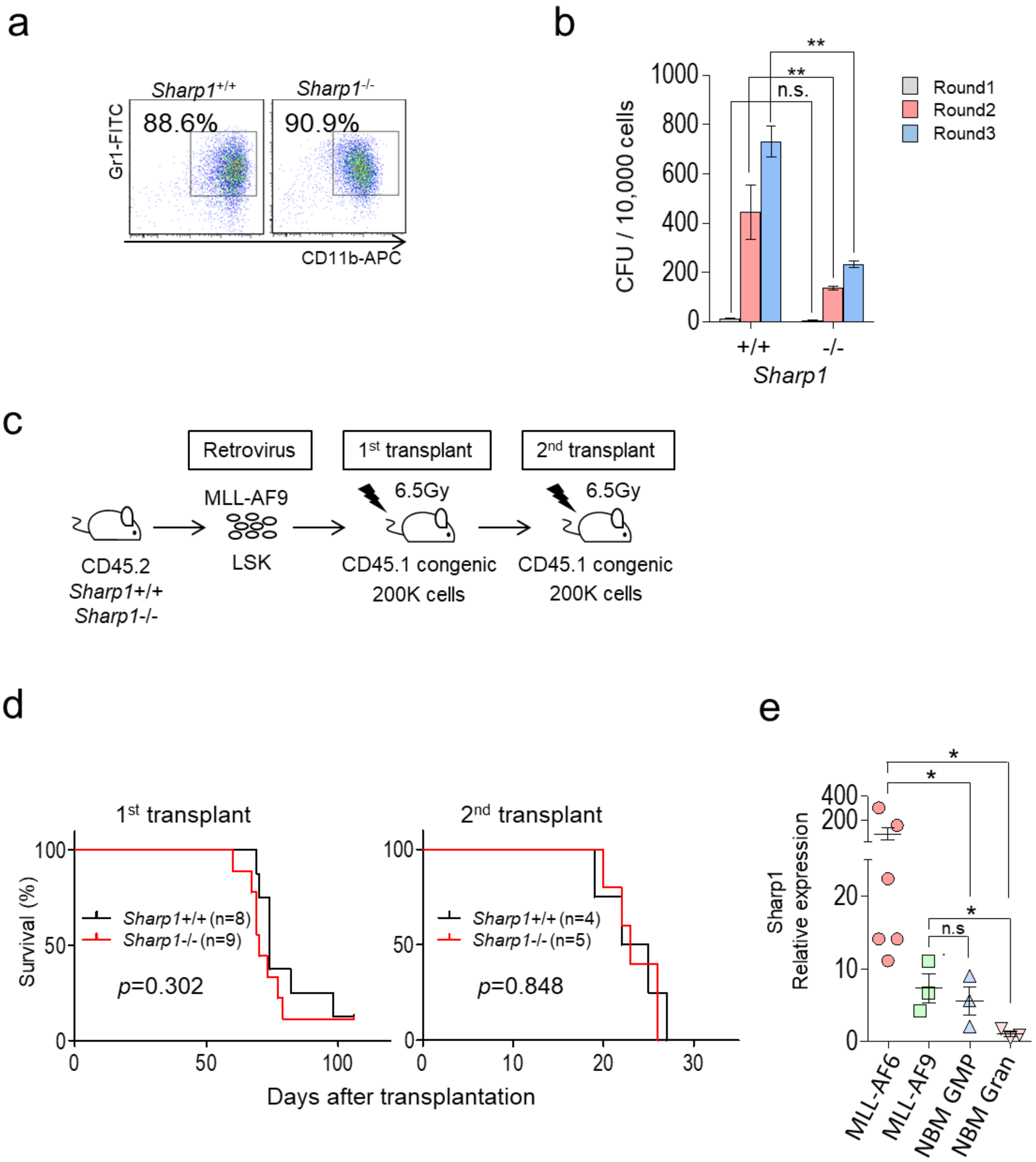
a
Sharp1+/+
Sharp1-/-
88.6%
90.9%
Gr1-FITC
CD11b-APC
b
CFU / 10,000 cells
1000
800
600
400
200
0
**
**
n.s.
Round1
Round2
Round3
+/+
-/-
Sharp1
c
Retrovirus
1st transplant
2nd transplant
MLL-AF9
6.5Gy
6.5Gy
LSK
CD45.2
Sharp1+/+
Sharp1-/-
CD45.1 congenic
200K cells
CD45.1 congenic
200K cells
d
1st transplant
2nd transplant
Survival (%)
100
50
0
Sharp1+/+ (n=8)
Sharp1-/- (n=9)
p=0.302
Sharp1+/+ (n=4)
Sharp1-/- (n=5)
p=0.848
0
50
100
0
10
20
30
Days after transplantation
e
Sharp1
Relative expression
400
200
20
10
0
*
*
*
n.s
MLL-AF6
MLL-AF9
NBM GMP
NBM Gran

Supplementary Figure 4. Related to Figure 4

(a) Representative flow cytometry plot for Gr1 and CD11b of leukemic BM cells. Percentage of $Gr1^{+}CD11b^{+}$ cells is shown. (b) Number of colonies per 10,000 cells observed 7 days after each replating of MLL-AF6 transduced *Sharp1*$^{+/+}$ and *Sharp1*$^{-/-}$ LSK cells. The graph is a representative example of three independent experiments and presented as mean ± s.e.m. (c) Experimental strategy for development of MLL-AF9 AML in *Sharp1*$^{+/+}$ and *Sharp1*$^{-/-}$ mice. (d) Kaplan Meyer survival curve of sublethally irradiated congenic mice transplanted with 200,000 cells from (left panel) the first plate and (right panel) whole bone marrow cells isolated from leukemic recipients following the first transplant. *P* values were determined by Log-rank (Mantle-Cox) Test. (e) qPCR for Sharp1 mRNA expression in murine MLL-AF6 and MLL-AF9 AML cells, normal bone marrow GMP (NBM GMP) and normal bone marrow granulocytes (NBM Gran, $Gr1^{+}CD11b^{+}$ cells from C57BL/6 mice). Values relative to the average expression of NBM Gr are shown. *P* values were determined by Mann–Whitney U test.

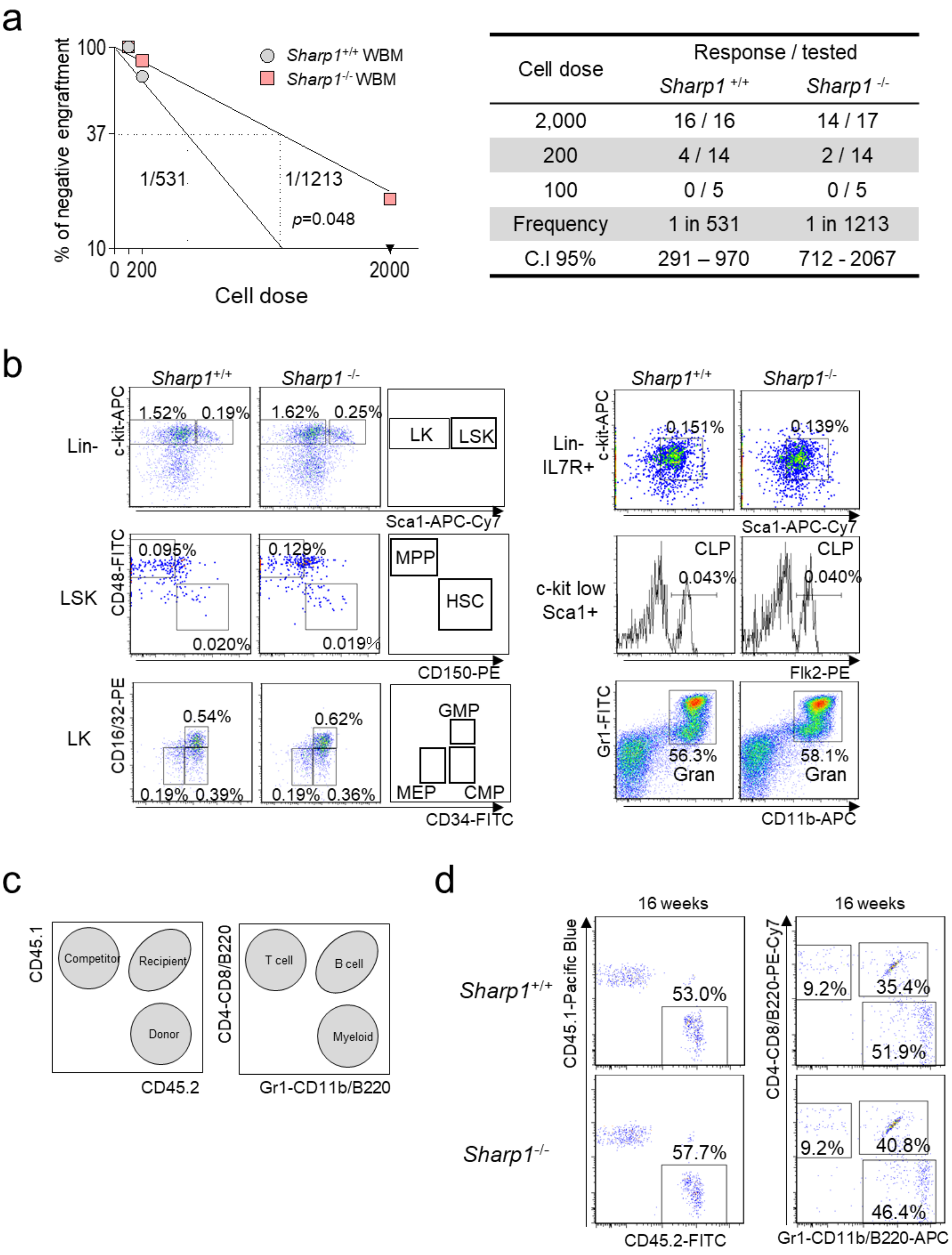

Cell dose	Response / tested	
	Sharp1 +/+	*Sharp1* -/-
2,000	16 / 16	14 / 17
200	4 / 14	2 / 14
100	0 / 5	0 / 5
Frequency	1 in 531	1 in 1213
C.I 95%	291 – 970	712 - 2067

Supplementary Figure 5. Related to Figure 5

(a) Limiting Dilution Assay (LDA). The indicated numbers of leukemic whole bone marrow (WBM) cells (ML-AF6 AML *Sharp1*$^{+/+}$ and *Sharp1*$^{-/-}$) were transplanted into sublethally irradiated congenic mice. Inverted triangle represents 0% of negative engraftment of *Sharp1*$^{+/+}$ WBM cells. (b) Representative flow cytometry plots for HSPC and mature myeloid cells from age and gender-matched *Sharp1*$^{+/+}$ and *Sharp1*$^{-/-}$ mice. Percentages of the gated populations are shown. (c) Cartoon showing the chimerism analysis of peripheral blood by flow cytometry. When staining for CD45.1 and CD45.2, CD45.1 and CD45.2 double positive cells are recipient-derived cells. CD45.2 only positive cells are donor-derived cells, while CD45.1 only positive cells are competitor-derived cells. When staining for B220/CD4-CD8 and CD11b-Gr1/B220, the double positive population is B220^{+} B lineage cells. CD4-CD8 only positive are T lineage cells, while CD11b-Gr1 only positive cells are from the myeloid lineage. (d) Representative flow cytometry plots for identification of donor cell, myeloid, B and T cell in peripheral blood of recipients of *Sharp1*$^{+/+}$ (upper) and *Sharp1*$^{-/-}$ mice (lower) 16 weeks after transplantation. Percentages of the gated populations are shown.

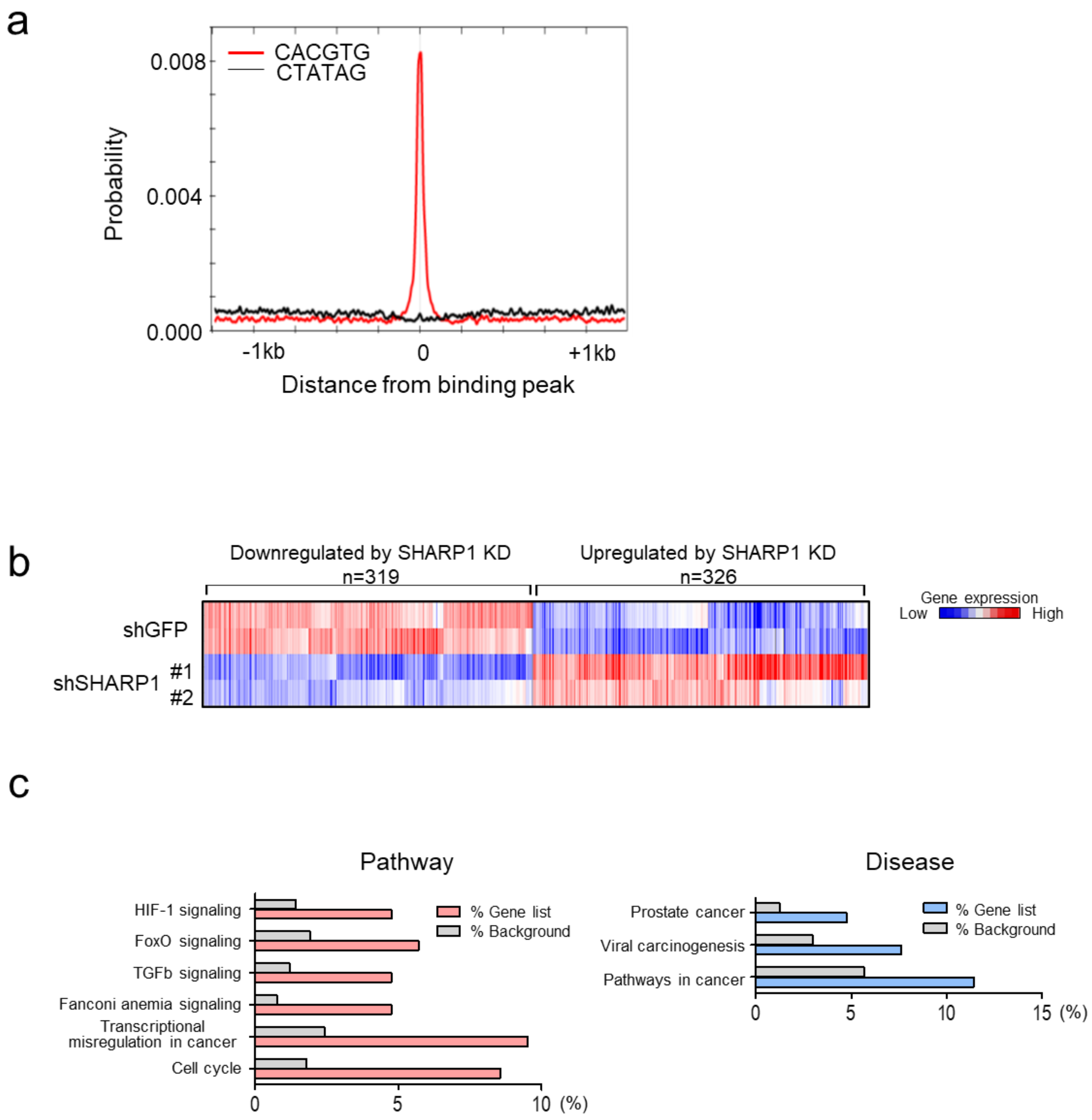
a
CACGTG
CTATAG
0.008
0.004
0.000
Probability
-1kb
0
+1kb
Distance from binding peak
b
Downregulated by SHARP1 KD
n=319
Upregulated by SHARP1 KD
n=326
shGFP
shSHARP1
#1
#2
Gene expression
Low
High
c
Pathway
HIF-1 signaling
FoxO signaling
TGFb signaling
Fanconi anemia signaling
Transcriptional misregulation in cancer
Cell cycle
% Gene list
% Background
0
5
10 (%)
Disease
Prostate cancer
Viral carcinogenesis
Pathways in cancer
% Gene list
% Background
0
5
10
15 (%)

d

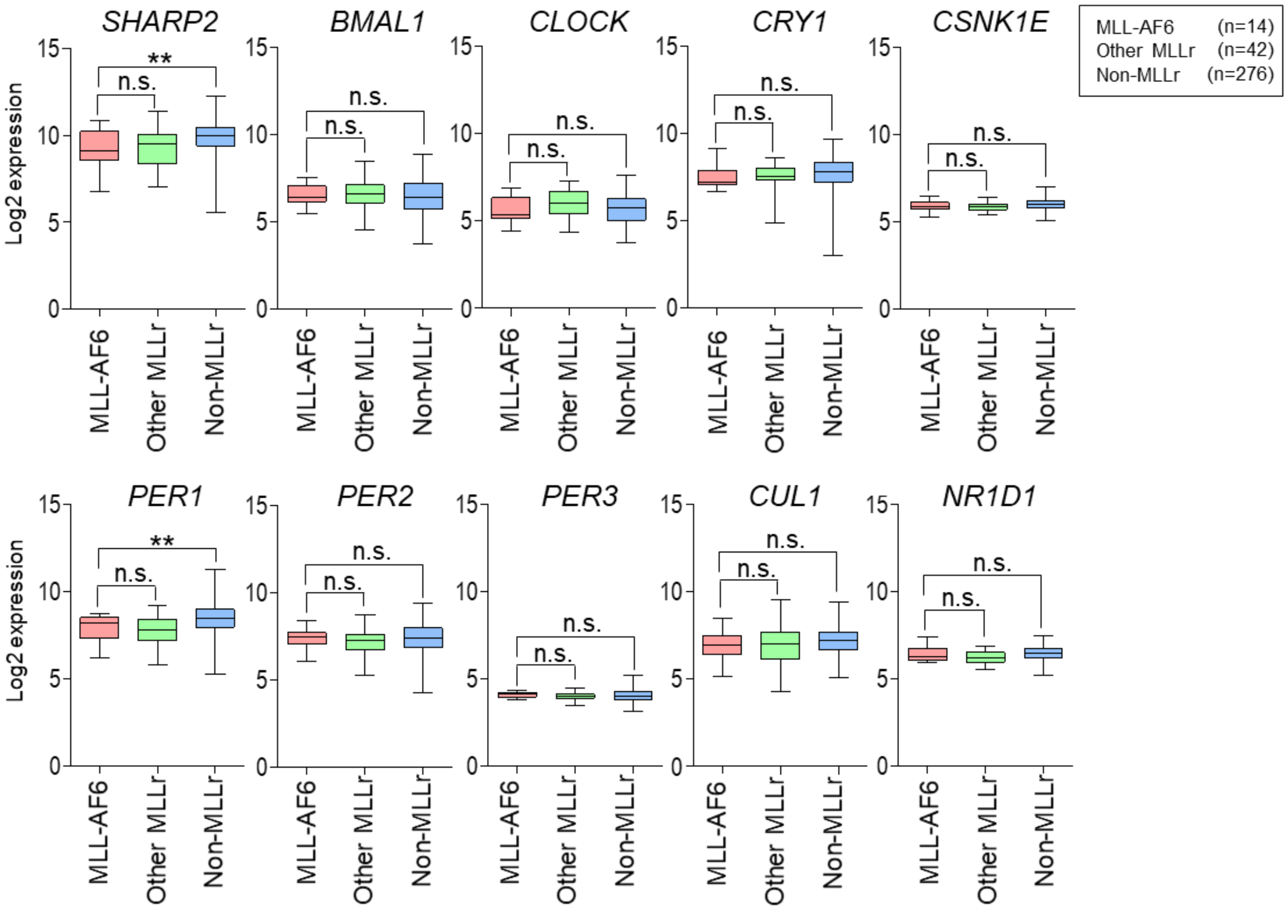
SHARP2
BMAL1
CLOCK
CRY1
CSNK1E
PER1
PER2
PER3
CUL1
NR1D1
MLL-AF6 (n=14)
Other MLLr (n=42)
Non-MLLr (n=276)
Log2 expression
n.s.
**
MLL-AF6
Other MLLr
Non-MLLr
0
5
10
15

e

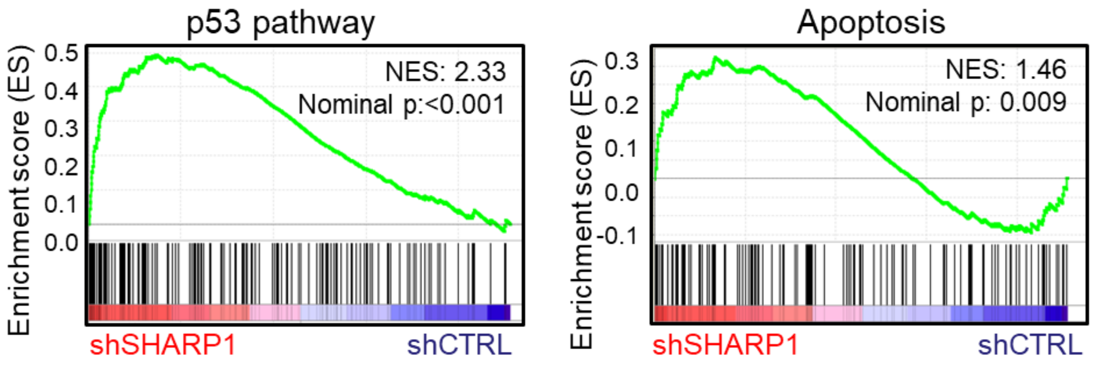
p53 pathway
NES: 2.33
Nominal p:<0.001
Apoptosis
NES: 1.46
Nominal p: 0.009
Enrichment score (ES)
shSHARP1
shCTRL

Supplementary Figure 6. Related to Figure 6

(a) Distributions of *cis* elements around the SHARP1 binding sites with CACGTG motif compared to control CTATAG motif. (b) Heatmap images representing the relative expression levels of 319 SHARP1-bound genes downregulated and 326 SHARP1-bound genes upregulated by SHARP1 knockdown (KD) in ML-2 cells. Each row corresponds to a gene and is normalized across the row. (c) Pathway analysis of SHARP1-bound genes downregulated by SHARP1 KD. (d) Box plot showing the average expression of circadian clock genes in AML patients. Other MLLr: MLL-rearranged AML other than MLL-AF6 AML, Non-MLLr: non MLL-rearranged AML. Gene expression data of patients were obtained from GSE19577, GSE14468 and GSE61804. Box plot extends from the 25th to 75th percentiles and the whisker extends from the minimum level to the maximum. Median value is plotted in the box. (e) Enriched gene sets in ML-2 shSHARP1 cells over shGFP: p53 pathway (HALLMARK P53 PATHWAY) and Apoptosis (HALLMARK APOPTOSIS).

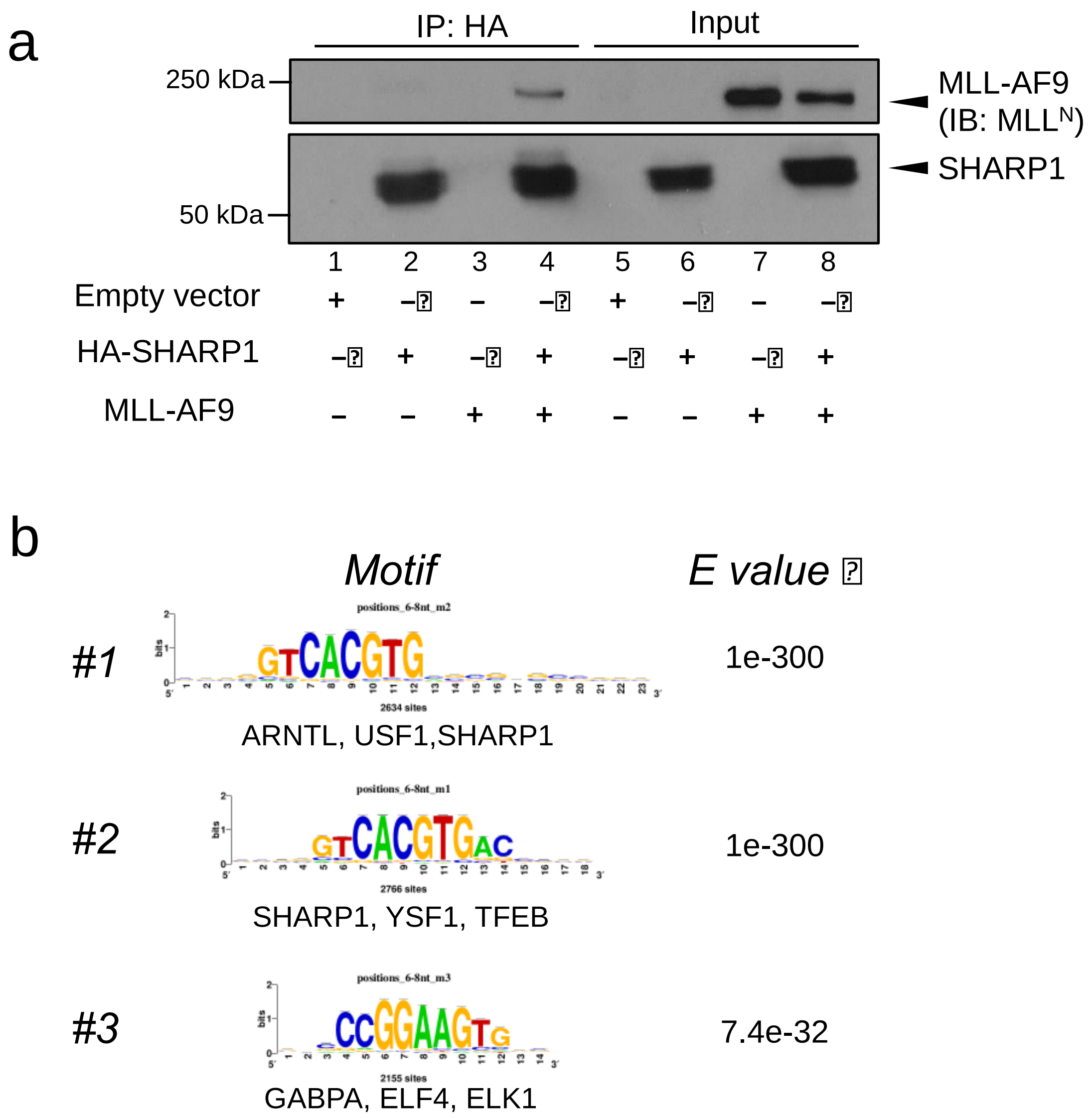

Supplementary Figure 7. Related to Figure 7.

(a) Co-immunoprecipitation studies of SHARP1 and MLL-AF9 with an anti-HA antibody in 293T cells transfected with plasmids encoding MLL-AF9 and/or HA-tagged SHARP1. Proteins present in immunoprecipitates (IP, lane 1-4) or whole cell lysates of transfected cells (input, lane 5-8) were separated by SDS-PAGE and immunoblotted with antibodies specific for MLL^N and SHARP1. Interaction of SHARP1 and MLL-AF9 was detected (lane 4) and not observed in negative control lanes with either empty vector, SHARP1 or MLL-AF9 only (lane 1-3). Western blots are representative of three independent experiments (b) Top 3 enriched motifs within SHARP1 ChIP-seq peaks on gene promoters revealed by peak-motifs module from the RSAT suite, using oligomer length ranging from 6 to 8 nucleotides and the “merge lengths for assembly” option.

Figure 2g

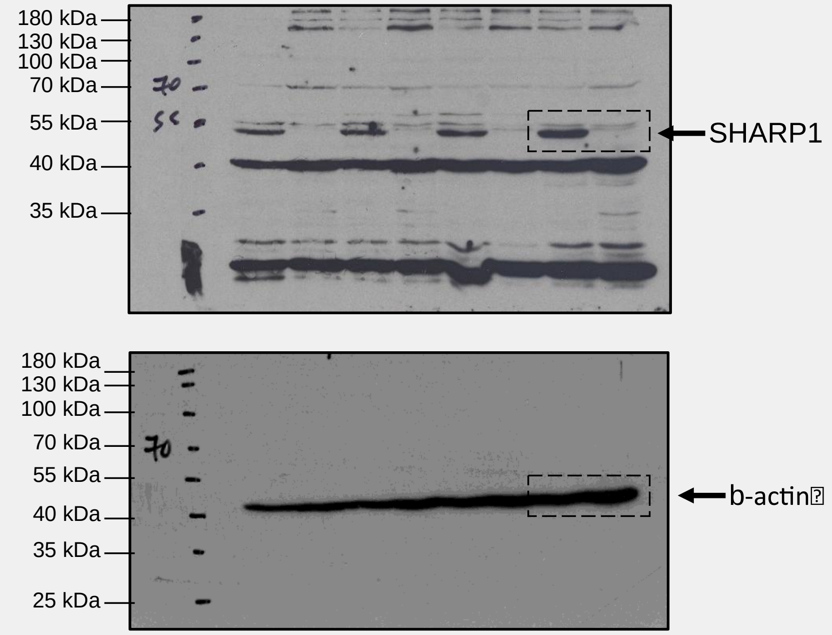

Figure 2e

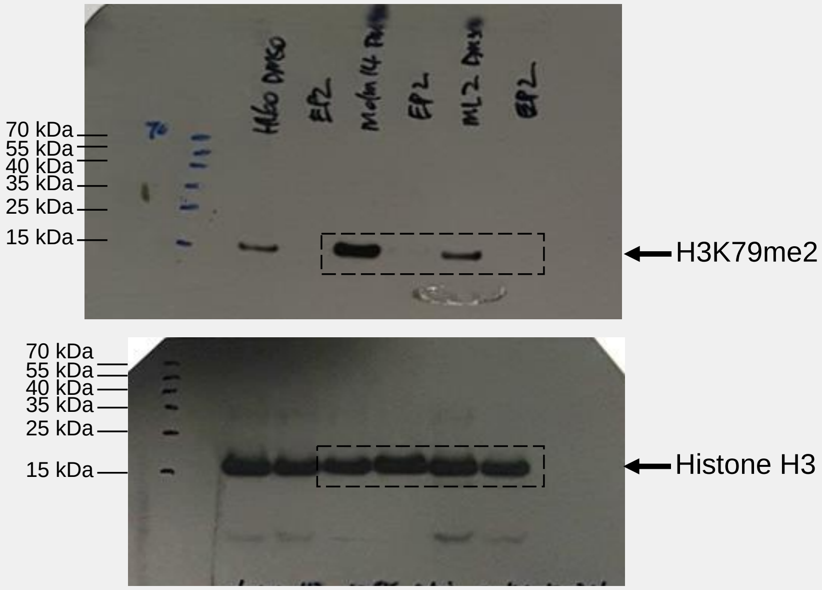

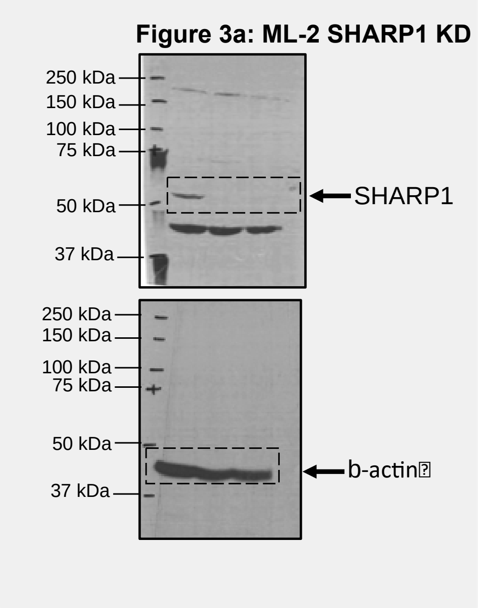
Figure 3a: ML-2 SHARP1 KD
250 kDa
150 kDa
100 kDa
75 kDa
50 kDa
37 kDa
SHARP1
250 kDa
150 kDa
100 kDa
75 kDa
50 kDa
37 kDa
b-actin

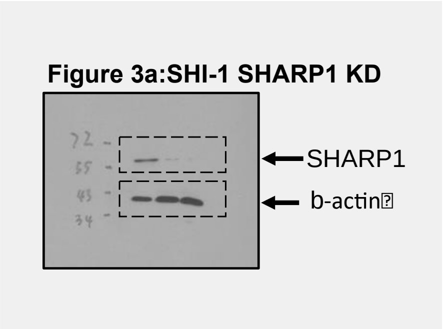
Figure 3a:SHI-1 SHARP1 KD
SHARP1
b-actin

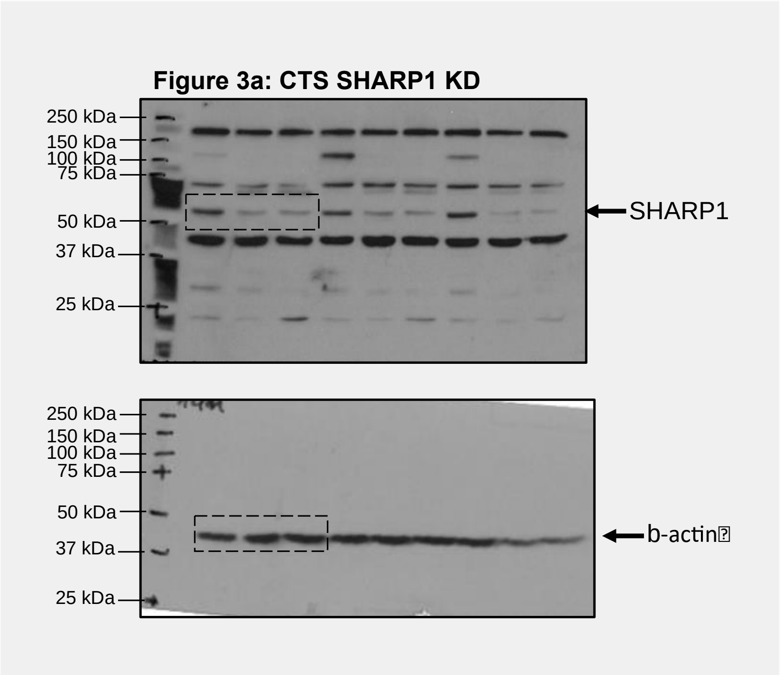
Figure 3a: CTS SHARP1 KD
250 kDa
150 kDa
100 kDa
75 kDa
50 kDa
37 kDa
25 kDa
SHARP1
250 kDa
150 kDa
100 kDa
75 kDa
50 kDa
37 kDa
25 kDa
b-actin

Figure 7e

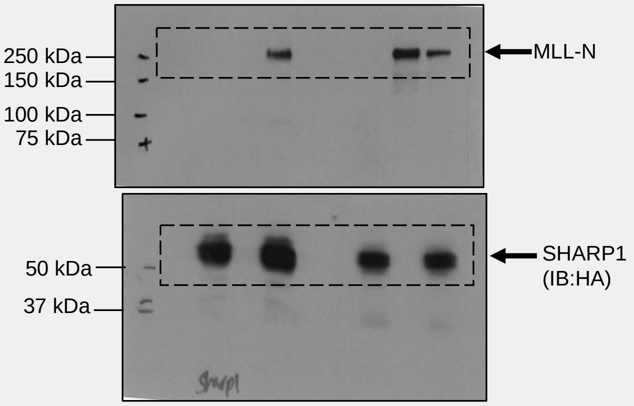

Figure 7g

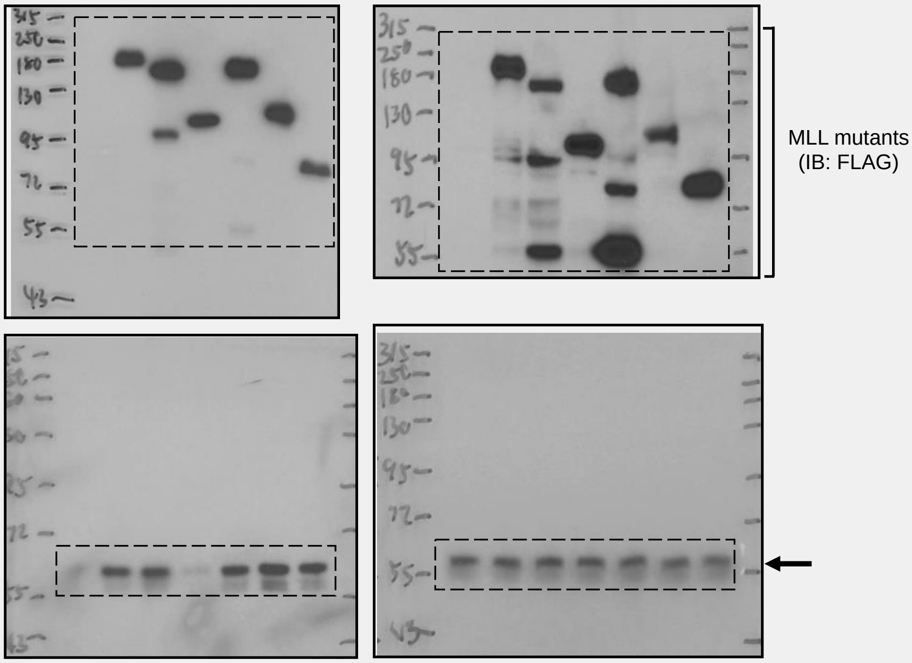

Supplementary Figure 7a

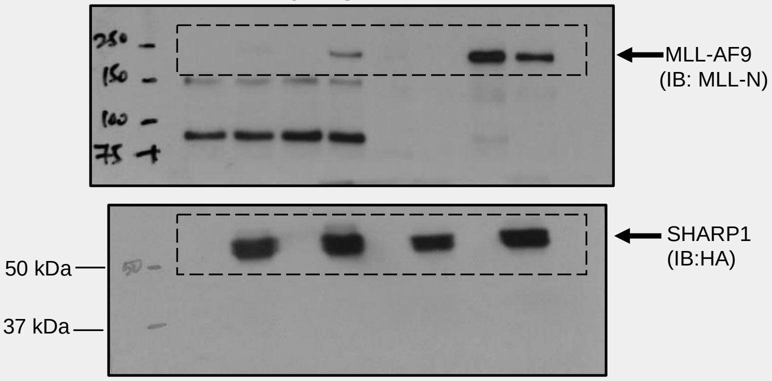

Supplementary Figure 8. Uncropped Western blot images from Figures 2, 3, 7 and Supplementary Figure 7.

Shown are the uncropped Western blot images presented in the main and supplementary figures. Images are labeled corresponding to their figures from the manuscript, with the respective data denoted by dotted boxes. All data are representative of at least three independent experiments.

Supplementary Table 1. MLL-AF6 bound genes in human MLL-AF6 AML cells

ADAMTS19-AS1	*DLX5*	*JMJD1C*	*MMRN1*	*SKIDA1*
ANO6	*DLX6*	*JMJD1C-AS1*	*MYB*	*SLC35D1*
ANXA2R	*DLX6-AS1*	*KLRF2*	*NPAS3*	*SSPN*
APOLD1	*DOCK8*	*LINC00938*	*NSMAF*	*SUPT3H*
ARID2	*EIF4E3*	*LOC100130992*	*P2RY1*	*SYDE2*
BAZ2B	*EMB*	*LOC100132356*	*PARP8*	*TAPT1*
BHLHE41	*FAM169A*	*LOC100499489*	*PDCD6IPP2*	*TAPT1-AS1*
BMI1	*FLJ32255*	*LOC100506159*	*PGM5P3-AS1*	*TCF4*
C9orf66	*FOXD4L1*	*LOC100996255*	*PGM5P4-AS1*	*TCTEX1D1*
CDK13	*FOXP1*	*LOC153684*	*PLEKHA8P1*	*TMEM117*
CDK6	*FRY*	*LOC643072*	*PRDM8*	*TRPS1*
CDKN1B	*FUT4*	*LOC646762*	*PROK2*	*WHAMMP2*
CDKN2C	*HOTTIP*	*LOC648987*	*PTPRK*	*WHAMMP3*
CHSY3	*HOXA10*	*MBNL1*	*RBMS1*	*ZNF521*
CLEC2B	*HOXA10-AS*	*MBNL1-AS1*	*REEP3*	*ZNRF2P1*
COMMD3	*HOXA10-HOXA9*	*MCOLN3*	*RNF220*	*ZNRF2P2*
COMMD3-BMI1	*HOXA11*	*MEF2C*	*RUNX2*	
CPEB2	*HOXA11-AS*	*MEF2C-AS1*	*SATB1*	
DACH1	*HOXA13*	*MIR196B*	*SATB1-AS1*	
DHRS7	*HOXA7*	*MIR4785*	*SCAF11*	
DLEU1	*HOXA9*	*MIR591*	*SENP6*	

List of 101 MLL^N-bound genes in ML-2 cells from ChIP-seq analysis.

Supplementary Table 2. MLL-AF6 target genes in human MLL-AF6 AML cells

ADAMTS19-AS1	*DLX5*	*KLRF2*	*MYB*	*SKIDA1*
ANO6	*DLX6*	*LINC00938*	*NPAS3*	*SLC35D1*
ANXA2R	*DLX6-AS1*	*LOC100130992*	*NSMAF*	*SSPN*
APOLD1	*DOCK8*	*LOC100132356*	*P2RY1*	*SUPT3H*
ARID2	*EIF4E3*	*LOC100499489*	*PARP8*	*SYDE2*
BAZ2B	*EMB*	*LOC100506159*	*PDCD6IPP2*	*TAPT1*
BHLHE41	*FAM169A*	*LOC100996255*	*PGM5P3-AS1*	*TAPT1-AS1*
BMI1	*FLJ32255*	*LOC153684*	*PLEKHA8P1*	*TCF4*
C9orf66	*FOXD4L1*	*LOC643072*	*PRDM8*	*TCTEX1D1*
CDK13	*FOXP1*	*LOC646762*	*PROK2*	*TMEM117*
CDK6	*FRY*	*LOC648987*	*PTPRK*	*TRPS1*
CDKN2C	*FUT4*	*MBNL1*	*RBMS1*	*WHAMMP2*
CHSY3	*HOTTIP*	*MBNL1-AS1*	*REEP3*	*WHAMMP3*
CLEC2B	*HOXA10*	*MCOLN3*	*RNF220*	*ZNF521*
COMMD3	*HOXA11*	*MEF2C*	*RUNX2*	*ZNRF2P1*
CPEB2	*HOXA7*	*MEF2C-AS1*	*SATB1*	*ZNRF2P2*
DACH1	*HOXA9*	*MIR4785*	*SATB1-AS1*	
DHRS7	*JMJD1C*	*MIR591*	*SCAF11*	
DLEU1	*JMJD1C-AS1*	*MMRN1*	*SENP6*	

List of 92 MLL + H3K79me2 overlapped genes in ML-2 cells from ChIP-seq analysis.

Supplementary Table 3. MLL-AF6 and SHARP1 co-target genes in human MLL-AF6 AML cells

ADAMTS19-AS1	DLEU1	LINC00938	NPAS3	SKIDA1
ANO6	DLX6	LOC100130992	NSMAF	SLC35D1
ANXA2R	DOCK8	LOC100132356	P2RY1	SSPN
APOLD1	EMB	LOC100499489	PARP8	SUPT3H
ARID2	FAM169A	LOC100506159	PDCD6IPP2	SYDE2
BAZ2B	FLJ32255	LOC100996255	PGM5P3-AS1	TAPT1
BHLHE41	FOXD4L1	LOC153684	PRDM8	TCF4
BMI1	FOXP1	LOC646762	PROK2	TCTEX1D1
CDK13	FRY	LOC648987	PTPRK	TMEM117
CDK6	FUT4	MBNL1	REEP3	TRPS1
CDKN2C	HOXA10	MBNL1-AS1	RNF220	WHAMMP2
CHSY3	HOXA11	MEF2C	RUNX2	WHAMMP3
CLEC2B	HOXA7	MEF2C-AS1	SATB1	ZNF521
CPEB2	HOXA9	MIR4785	SATB1-AS1	ZNRF2P1
DACH1	JMJD1C	MIR591	SCAF11	
DHRS7	KLRF2	MYB	SENP6	

List of 78 MLL + H3K79me2 + SHARP1 overlapped genes in ML-2 cells from ChIP-seq analysis.

Supplementary Table 4. Primary antibodies for FACS

Antigen	Species	Clone	Cat #	Company	Dilution	Fluorochrome
CD11b	mouse	M1/70	17-0112-82	eBioscience	1:200	APC
CD11b	mouse	M1/70	25-0112-82	eBioscience	1:200	PE-CY7
Gr1/Ly6G	mouse	RB6-8C5	11-5931-82	eBioscience	1:200	FITC
Gr1/Ly6G	mouse	RB6-8C5	25-5931-82	eBioscience	1:200	PE-CY7
CD3e	mouse	145-2C11	25-0031-82	eBioscience	1:200	PE-CY7
CD4	mouse	GK1.5	25-0041-82	eBioscience	1:200	PE-CY7
CD8a	mouse	53-6.7	25-0081-82	eBioscience	1:200	PE-CY7
B220/CD45R	mouse	RA3-6B2	553092	BD Pharmingen	1:100	APC
B220/CD45R	mouse	RA3-6B2	25-0452-82	eBioscience	1:100	PE-CY7
CD19	mouse	1D3	25-0193-82	eBioscience	1:100	PE-CY7
Ter119	mouse	TER-119	25-5921-82	eBioscience	1:100	PE-CY7
NK1.1	mouse	145-2C11	25-5941-82	eBioscience	1:200	PE-CY7
CD117/c-kit	mouse	2B8	553356	BD Pharmingen	1:100	APC
Sca-1/Ly-6A/E	mouse	D7	560654	BD Pharmingen	1:100	APC-CY7
CD150 (SLAM)	mouse	TC15-12F12.2	115904	Biolegend	1:100	PE
CD48	mouse	HM48-1	11-0481-82	eBioscience	1:100	FITC
CD34	mouse	RAM34	553733	BD Pharmingen	1:50	FITC
CD16/32	mouse	2.4G2	553145	BD Pharmingen	1:100	PE
CD127/IL7Ra	mouse	A7R34	11-1271-85	eBioscience	1:100	FITC
CD135/Flt3	mouse	A2F10	25-0452-82	eBioscience	1:100	PE
CD45.1	mouse	A20	110721	Biolegend	1:200	Pacific Blue
CD45.2	mouse	104	109805	Biolegend	1:200	FITC
CD11b	human	ICRF44	550019	BD Pharmingen	1:200	APC
CD14	human	M5E2	555398	BD Pharmingen	1:200	PE
CD15	human	HI98	551376	BD Pharmingen	1:200	APC

Supplementary Table 5. Primary and secondary antibodies for Western blotting

Antigen	Company	Catalog no.	Dilution
SHARP-1	Santa Cruz Biotechnology	H-72	1:1000
H3K79me2	Abcam	ab3594	1:1000
Histone H3	Cell Signaling Technology	#9715	1:5000
MLL	Abcam	ab25735	1:100
β-actin	Santa Cruz Biotechnology	sc-47778	1:5000
FLAG	Sigma	F1804	1:10000
HA	Santa Cruz Biotechnology	sc-7392	1:2000
secondary anti-rabbit IgG	Cell Signaling Technology	#7074	1:2000
secondary anti-mouse IgG	Santa Cruz Biotechnology	sc-358914	1:2000
